# Manchester stands united: Place‐based identity facilitates resilience in the aftermath of a mass emergency

**DOI:** 10.1111/bjso.70056

**Published:** 2026-02-17

**Authors:** Helen Hart, Clifford Stevenson, Blerina Kellezi

**Affiliations:** ^1^ University of Salford Manchester UK; ^2^ Nottingham Trent University Nottingham UK; ^3^ Tohoku University Sendai Japan

**Keywords:** community resilience, disasters, place identity, shared identity, social identity, terrorist attack

## Abstract

Understanding community resilience to disasters is fundamentally important in a world characterized by increasing political and environmental instability. The Social Identity Model of Collective Resilience has examined how the shared identity that emerges among neighbourhood residents affected by disasters can facilitate and coordinate effective collective responses, but has yet to examine impacts on community members beyond those directly affected. This is particularly important given the role of social identities in creating shared vulnerability and resilience to collective trauma among those indirectly affected, as well as evidence that neighbourhood identification can provide residents with collective resilience to a range of shared socio‐economic and environmental stressors. The present study addresses this gap through an exploration of residents' accounts of the occurrence and aftermath of a terrorist attack on Manchester, England in 2017. The thematic analysis of retrospective interviews with 18 city residents indirectly affected by the bomb revealed that two key aspects of Mancunian identity – diversity and endurance of the city – were used to interpret the event and reported to facilitate coordinated coping and collective recovery. The implications are that identifying and enhancing local norms of cohesion and endurance can play a part in providing communities with resilience to future disasters.

## INTRODUCTION

### Community resilience to disasters

Research on disasters has typically focussed upon natural disasters, which is unsurprising given the extent of such occurrences. For the purpose of this article, disaster is defined as a ‘*potentially traumatic event that is collectively experienced, has an acute onset, and is time delimited*’ (McFarlane & Norris, 2006, p. 15). This definition encompasses acts of nature and human‐made disasters, such as terrorism but excludes pandemics, chronic environmental hazards and war.

Research on disaster resilience consistently highlights the role of social relationships and social capital as critical protective factors. Strong social bonds enable communities to mobilize resources, share information and provide mutual aid during crises. Aldrich (2012) argues that communities with strong bonding and bridging ‘social capital’ recover more quickly after disasters because these ties foster cooperation and access to support. Norris et al. (2008) similarly identify social connectedness as a core component of ‘community resilience’ (defined as ‘*a process linking a set of adaptive capacities to a positive trajectory of functioning and adaptation after a disturbance*’ *2008, p. 130*), underpinning both emotional coping and practical assistance.

Empirical studies of Hurricane Katrina show that residents relied on bonding ties for immediate survival and bridging and linking ties for long‐term recovery, with social networks often outperforming formal institutions in delivering aid and sustaining morale (e.g. Hawkins & Maurer, 2010). Likewise, research on the 2011 Japanese tsunami demonstrates that communities with dense social networks and strong social infrastructure—such as community centres and local associations—experienced lower mortality rates and faster recovery, underscoring that resilience stems from social connections rather than physical preparedness alone (e.g. Aldrich, 2023).

In terms of the social psychological processes underpinning effective reactions to disasters and emergencies, social identity processes have been found to be pivotal in the public's responses to disasters and emergencies (Drury, 2012). Against a background of media and government policy assumptions that the general public typically freezes, panics or engages in criminal behaviour during a mass emergency, studies of a range of disasters (Drury et al., 2009) show that public responses are typically characterized by helping behaviour and coordinated efforts to respond to the emergency.

The Social Identity Model of Collective Resilience (SIMCR; Drury et al., 2019) was developed to explain how this shared coping response emerges during an emergency or disaster. Careful analyses of survivors' accounts of these events showed how people typically realize that they share a common fate with others directly affected by the emergency. This gives rise to a shared identity or ‘we‐ness’ among those co‐present, which in turn leads to the belief that the group shares common goals. Shared identity is also associated with increased trust, helping behaviour and the acceptance of help in the spirit it is offered (Haslam et al., 2012) which facilitates coordinated behaviours among survivors. Thus, from a group of unacquainted strangers, a cohesive group emerges which is capable of mutual assistance and working together towards common objectives. Moreover, the feeling of shared identity predicts longer term recovery after such events (Ntontis et al., 2017).

While SIMCR primarily focuses on the identities emerging from emergencies and disasters, it also acknowledges that pre‐existing community structures and relationships are often important in crises which affect residential areas (Ntontis et al., 2020). Drury et al. (2019) advise that government agencies set up local community groups based on meaningful engaging identities to scaffold group processes ahead of an emergency. To do so, they advocate understanding the norms and values of local communities, so that they can work with, rather than against these norms in the event of a disaster.

However, SIMCR has yet to focus on how different enduring understandings of community identity can shape responses. Furthermore, SIMCR and other approaches which focus on immediate responses to disasters and emergencies typically focus on those directly affected by the events rather than the wider population of local communities who are indirectly affected and are also a potential source of support and coping. Relatedly, SIMCR is primarily focussed on the immediate occurrence and aftermath of disasters and less on the long‐term consequences and coping processes among those affected and their wider networks. To consider these more distal group processes, we now turn to other research which seeks to explain why emergencies and disasters affect wider communities beyond those immediately affected and how chronic social identity processes among residential communities can help them cope with disasters.

### Group‐based experiences of, and responses to trauma

The Social Identity Model of Traumatic Identity Change (SIMTIC) argues that psychology has typically failed to recognize the collective dimensions of the experiences of and reactions to aversive experiences (Muldoon & Lowe, 2012). Research in this tradition demonstrates that pre‐existing social identities shape both exposure to danger and its interpretation, as group memberships determine who is most at risk as well as how threat is appraised. For example, minority and disadvantaged groups often face greater exposure to political violence and perceive such events as more distressing (Muldoon & Trew, 2000; Schmid & Muldoon, 2015). Conversely, strong occupational or activist identities can normalize extreme risk, framing it as routine or meaningful (Haslam et al., 2005). In effect, threat is never neutral and is filtered through the lens of identity and collective experience (Këllezi & Reicher, 2012; Muldoon & Lowe, 2012).

Second, this work indicated that trauma extends beyond direct victims. DSM‐5 recognizes indirect exposure, such as learning of harm to loved ones or encountering traumatic material professionally as sufficient for PTSD diagnosis (American Psychiatric Association, 2013; Breslau & Kessler, 2001). Evidence shows that witnessing violence or hearing about atrocities can evoke severe stress responses, particularly when these events threaten valued relationships (Charuvastra & Cloitre, 2008). Human‐designed traumas like rape or terrorism are especially harmful because they violate social norms and erode trust, amplifying vulnerability even among those indirectly affected (Kellezi & Reicher, 2014; Shalev & Freedman, 2005).

Third, long‐term social identities often protect against trauma's psychological toll. While shared trauma can generate new identities, maintaining pre‐existing valued identities provides stability and meaning, reducing PTSD risk (Muldoon & Downes, 2007). Trauma can also strengthen existing collective identities, enabling post‐traumatic growth (Muldoon et al., 2017). However, when trauma stigmatizes identities, as with sexual violence, access to support diminishes, which intensifies distress (Këllezi et al., 2009; Këllezi & Reicher, 2012).

SIMTIC therefore provides an explanation of how and why trauma is experienced indirectly by group members beyond those immediately affected as well as how wider group processes can aid recovery. In relation to emergencies and disasters, there is some support for shared identity processes underpinning both collective vulnerability and coping with traumatic incidents. The Paris 2015 attacks associated the expression of emotion and solidarity well beyond those directly affected (Garcia & Rimé, 2019) and residents' collective memory of dealing with previous disasters helped to shape current responses (Farny & Dentoni, 2025; Monteil et al., 2020). However, to understand how local community identity structures perception and reaction to chronic threat and stress, which can have significant implications for their ability and willingness to support direct survivors, we need to consider the specific literature on this topic.

### Local community identity and collective coping

Local community identity has been shown to constitute both a source of collective vulnerability and strength to residents. Research by McNamara and colleagues (McNamara et al., 2013) showed how residents of disadvantaged communities in Limerick, Ireland evidenced resilience to economic challenge. To the extent that they shared a strong identity with other members of the community, they felt a sense of ‘collective efficacy’ of ability to collectively cope with the challenges faced by their communities, which in turn predicted their well‐being (see also Fong et al., 2019; Heath et al., 2017). However, stigmatization of the community's identity was found to undermine these collective resilience processes (McNamara et al., 2013; Stevenson et al., 2014). In other words, both the strength and the meaning of local community identity are important in shaping community perception of and resilience to economic threats.

In terms of social threats, a further series of studies have shown that community identification also reduces the negative impact of residential diversification. Stevenson and colleagues have demonstrated that within ethnically and religiously divided neighbourhoods, an influx of outgroup members is experienced less negatively if the resident feels a strong bond of attachment to their local area, which in turn predicts lower intergroup anxiety (Stevenson & Sagherian‐Dickey, 2018, Stevenson, McNamara, et al., 2019, Stevenson, Easterbrook, et al., 2019, Stevenson et al., 2020). The positive reception of outgroup members does, however, depend upon the understanding of the local area as amenable to diversification, such that the meaning of the local identity shapes the reception of newcomers (Stevenson & Sagherian‐Dickey, 2018).

Community identification has also been shown to provide resilience to environmental factors including cold and noise. For example, the shared identity of pilgrims at the north Indian Magh Mela enabled them to cope with the harsh environmental conditions in a variety of ways. In terms of the cold, pilgrims understood the endurance of low temperatures to be part of their participation in the festival and consequently they willingly and enthusiastically embraced activities such as night‐bathing in freezing water (Pandey et al., 2014). Likewise, they experienced the high levels of unceasing ambient cacophonic noise at the festival as uplifting and peaceful, but only insofar as it accorded with their shared identity within the pilgrim community (Shankar et al., 2013).

However, community identification can constitute a risk factor when the individual is excluded from the community or when community identity is negative or stigmatized. Këllezi et al. (2009, 2012) labelled these group‐based risk factors as ‘social curse’ when investigating the impact of the Kosova war. These included the increased threat of the war experiences (primary appraisal) when they were perceived to violate community norms (e.g. man's failure to protect their families or fight for the national cause), and the role that perceived norm violation and stigma had on undermining community support (expectations, asking and giving of support) and effective coping strategies to deal with the war experiences. Relatedly, McNamara and colleagues showed that perceptions of community stigma deterred residents from engagement in collective action and help‐seeking behaviours (McNamara et al., 2013; Stevenson et al., 2014). Këllezi et al. (2023, 2025) have also shown that communities can undermine access to support, recognition and justice following traumatic experiences, leading to community divisions and conflict.

In effect, these studies indicate that community identity can transform the interpretation of social, economic and environmental threats into more (or less) manageable experiences and provide social resources to cope with these threats (Haslam et al., 2024). They also indicate that the content or meaning of the community's identity is pivotal in shaping this response, and that where an individual is excluded from the community these coping resources may be lost.

In sum, SIMCR documents the emergent identity processes among those experiencing an emergency and disaster, while SIMTIC indicates that the indirect impact of resultant trauma can affect the wider group. Moreover, research on local community identity shows the importance of local residential identification in providing or undermining the long‐term collective resilience of residents. However, research has yet to capture how local community identity processes shape the indirect experience of and response to a disaster among local residents. The present study takes this as its focus and seeks to explore firstly how a long‐standing local identity shapes the perception, experience and reaction to a collective disaster.

### Manchester and the 2017 arena bomb

Manchester is a city in the North‐West of England, United Kingdom. The metropolitan county of Greater Manchester is made up of 10 boroughs with a current population of ~2.7 million. It has an ethnically diverse community, with the UK Population Data for 2022 indicating the population as White (66.7%), Asian (17.1%), Black (8.6%), Mixed Race (4.7%), Arab (1.9%), Other (1.2%) (Bullen, 2015).

Bullen (2015) states that historically, Manchester has attracted immigrants on a large scale since the Industrial Revolution, with immigrants from Pakistan as the most populous group. As a consequence of its diversity, Manchester has a clearly observable multicultural social landscape, such as the ‘Curry Mile’ in South Manchester, with its many South‐Asian restaurants and shops, and one of the largest Chinatowns in Europe.

Historically, Manchester has had its share of adversity: from the Peterloo Massacre in 1819 when 18 people were killed as cavalry charged into a crowd of protesters (Hirsch, 2025) to the Manchester Blitz during the Second World War (Carter, 2015). More recently, the city has fallen victim to repeated terrorist attacks. On 15th June 1996, the Provisional Irish Republican Army (PIRA) left a truck containing a bomb in Manchester city centre outside a large shopping centre. The bomb caused significant structural damage and, although many people were injured, there were no fatalities (Jahangir, 2016).

It is within this context that the events which are the focus of the current research occurred. On 22nd May 2017, around 10 pm, a lone Mancunian male of Libyan ethnicity detonated a bomb in the foyer of Manchester Arena at the end of an ‘Ariana Grande’ concert, as people were leaving the event. Ariana Grande is a popular musician with a fan base of largely pre‐teen/early teenage females, so the Arena was attended by this age group and their parents at the time of the bomb. Twenty‐two people were killed by the blast and over a thousand people were injured (Watson, 2022). As well as physical injuries, French et al. (2019) through their research with the Manchester Resilience Hub highlighted the ongoing level of psychological distress among 3150 children, young people and adults affected by the bomb up to 12 months after. This was far‐reaching as many people who attended the event were from other parts of the country outside of the Manchester area.

Soon after the bomb, members of the community showed their respect to the victims and survivors by leaving various mementos and floral tributes around the city. These were later removed by different community organizations and repurposed to provide meaningful, long‐standing tributes such as compost made from the flowers for planting trees of hope. More than 10,000 tributes have been retained in the city to form the Manchester Together archive as a permanent record of the public response to the attack, which also includes a focus on the mass tattooing of the Manchester bee (Arvanitis, 2025).

The current study is interested in this collective response and explored how Manchester residents who were not directly affected by the bomb experienced these events, and how and why they responded in the ways that they did. In particular, it examined how the events were interpreted in relation to their identity as Mancunian and how their understandings of the city and its residents shaped their response to the bomb.

## MATERIALS AND METHODS

### Participants

Eighteen participants were recruited and interviewed by the researcher between February and July 2019 and participation in the research was entirely voluntary. This number of participants allowed for the inclusion of perspectives from multiple communities from within Manchester. It also became apparent through the interviews that participants were identifying similar experiences and attaching similar meanings to these experiences and responses to the events, so the interpretation of the story told by these participants was felt to be coherent and sufficient at that stage to cease further data collection. This is consistent with findings by Hennink et al. (2017).

Participants had to meet the criteria of being over the age of 20, resident in Greater Manchester (since 2014), and they must not have attended the Manchester arena concert at the time of the bomb or know anybody who was seriously harmed as a result of the bomb. These criteria were to ensure that all participants would have been over the age of 18 at the time of the bomb, had been living in Manchester for long enough prior to the bomb to be considered local residents, and could be considered general community members, rather than direct survivors of the bomb.

Stratified sampling was used to recruit participants from black and minority ethnic (BAME) communities within Greater Manchester which was aimed at capturing major variations rather than a common core (Patton, 2002). It was not possible to recruit additional participants from predominantly white, working‐class communities within the time frame of the study despite targeted efforts which were similar to those used to attract BAME participants in relevant communities. In addition, the researcher used personal and professional connections to try to recruit White, working‐class participants but none of those strategies were effective within the time frame.

Participant demographics are presented in Table 1. Interest in the research was initially gained via posts on LinkedIn and from word‐of‐mouth. Notices were also placed in libraries, GP practices, community cafes, community centres, places of worship and local shops asking for research participants.

**TABLE 1:** Participant demographics.

Code	Gender	Ethnicity	Age	Occupation	Education	Greater Manchester borough	Disability	Pseudonym
P1	F	White (British)	23	Student/Nursing Assistant	Undergrad	Bury	N	Anna
P2	F	White (E. European)	26	Student/Takeaway Manager	Undergrad	Salford	N	Bella
P3	F	White (British)	29	Student	Postgrad	Bolton	Y	Crystal
P4	M	White (British)	56	IT Company Director	Graduate	Stockport	N	Dave
P5	F	Chinese	49	Software engineer	Graduate	Stockport	N	Ella
P6	F	White (British)	47	Health & Safety Exec	PhD	Stockport	N	Fliss
P7	F	Asian (Pakistani)	21	Student	Undergrad	Manchester	N	Priti
P8	F	White (British)	73	Retired Teacher	College	Bolton	N	Georgia
P9	M	White (British)	68	Retired Surveyor	College	Bolton	Y	Harry
P10	F	White (British)	23	Library Assistant	Graduate	Wigan	N	Isla
P11	F	White (British)	33	Education Worker	Graduate	Bolton	Y	Julie
P12	M	White (British)	24	Student	Postgrad	Salford	N	Ken
P13	M	White (British)	32	Library Manager	College	Bolton	N	Lloyd
P14	F	White (British)	37	Arts/Theatre Manager	Graduate	Trafford	N	Mary
P15	F	Asian (Libyan)	36	Charity Worker	Graduate	Manchester	N	Amira
P16	M	Asian (Pakistani)	41	Solicitor	Postgrad	Manchester	N	Mo
P17	F	Asian (Pakistani)	58	Community Consultant	Graduate	Manchester	N	Nadia
P18	M	White (British)	58	Rabbi	Postgrad	Bury	N	Adam

### Materials

An interview guide was developed which covered seven broad areas and can be found in the Supplementary Material S1. Sample questions were as follows: personal information (describe how you fit in your local community), social identity (what are your own groups?), the bomb (what did you see regarding how your family were affected?), the immediate aftermath (what were the changes in processing this over time?), consequences in the present (what local changes have you seen?), hopes for the future (what can help Manchester into the future?) and final reflections based on what they discussed in relation to contributions from other participants.

The interview guide was initially piloted with two Manchester residents known to the first author. Changes were made following the first pilot to improve the accessibility of the questions.

### Procedure

Participants were interviewed in community locations, mainly in private rooms or in a quieter section of a community location, namely, a library and a community centre café. Full informed consent was obtained, and participants were advised of the withdrawal procedure. All participants were debriefed after their interviews. Sixteen participants were interviewed individually and two (a married couple) were interviewed together at their request. Steps were taken to ensure that both members of this couple had the opportunity to engage equally in the interview and the ways in which they questioned and probed each other's perspective during the interview were of use. Data were collected using a semi‐structured style of interview to understand themes from the participants' own perspective (Kvale, 2008).

### Ethics

Ethical approval for this research was given by Nottingham Trent University in November 2018. Participants did not receive any compensation. All aspects of this research were conducted in line with the Health and Care Professions Council's Standards of conduct, performance and ethics (, 2012) and the British Psychological Society's code of human research ethics (, 2014).

### Analytic approach

Transcripts were anonymized and any possible third‐party identifiers were removed. Each participant was allocated a pseudonym that was broadly reflective of their gender and ethnicity. Reflexive thematic analysis, as described by Braun et al. (2016), Braun & Clarke (2006, 2019) was the chosen method of analysis as patterns in relation to the research questions across the entire data set were of primary interest. Reflexive thematic analysis was undertaken through the theoretical lens of the social identity approach. The primary researcher led this six‐stage process through deep familiarization with the data set, first to assign meaning to participants' experiences and to ‘test out’ what made sense about their experiences. Second, initial coding of the data in line with existing theory as well as noting novel patterns from the participants was undertaken. It became apparent early on that some codes were repetitive or redundant, and others were too wide so these were reorganized so the third stage of theme development could begin. Initial themes were developed and reorganized to ensure they contained clearly defined concepts with enough data to illustrate each theme. Three themes were clearly identified at this point, in the areas of identity, coping and transforming. The process of analysis highlighted one participant (Priti) whose responses departed significantly from the others and these differences were incorporated into the analysis (Mauceri, 2013).

Through this process, sub‐themes became noticeable, and the fourth stage of reviewing themes was necessary to clearly assist with clarifying sub‐themes. The process of sub‐theme development was laborious and involved a constant state of refinement and cross‐referencing of data against each theme in order to ensure that themes and sub‐themes were clear, distinct and well evidenced, while holding in mind the experiences of the participants. The fifth stage of defining and naming themes was able to progress when themes and sub‐themes were clearly ordered and could tell the overall story of the research. Finally, all researchers agreed upon the clear selection of data extracts for each theme and analysis in order to present the interpretive story about the data.

## RESULTS

Three major themes were identified and analysed. These related to ‘Identity’, ‘Coping’ and ‘Transforming’. Themes and sub‐themes are presented in Table 2 and discussed in turn.

**TABLE 2:** Themes and sub‐themes.

Themes	Sub‐themes
1 ‘Manchester does have a special spirit’: Identity as a diverse and resilient city	
2 ‘You've got a hug of people around you’: Coping in response to the attack	2.1 Taking stock of the event 2.2 Helping behaviour 2.3 Grieving and tributes
3 ‘It takes the crap and makes it beautiful’: Transforming for the future	3.1 Restoration and hopefulness 3.2 Active belonging 3.3 Managing divisions and potential conflict

### Theme 1: ‘Manchester does have a special spirit’: Identity as a diverse and Resilient City

The participants reported that Manchester as a place, and ‘being Mancunian’ are clearly defined and meaningful. Being Mancunian is not tied to place of birth or race but characterizes residents' relationship with the city.

The overall context of Manchester as a place was referred to in positive terms that ranged from its industrial past, historical significance and creative heritage to its current ethnic diversity and technological advances. Participants appeared to know the city in depth and to take pride in it, despite being acutely aware of its socio‐economic problems. The relationship between the place and the people was evidenced by participants' accounts of how they came to be in Manchester, either through birth or through moving to the area, and how they felt a part of the fabric of the city:

Extract 1: Mary, white British woman, 30s

I had a really stable upbringing […] where I was brought up but I don't feel connected to there, I definitely feel Mancunian… Manchester lets people be adopted so people do allow that, but you also do need to say ‘I'm not really from here’ it's part of what people do but then once you've voiced that as long as you don't pretend its fine I think.

Mary refers to the expectation that as long as she made it clear that she was not originally from Manchester, she was still accepted as an ‘adopted’ Mancunian. Mary's reference to not pretending to be from the city, speaks to the need for authenticity within the community.

One participant had a very different perception of Manchester and her place in the city. Priti was a female student in her 20s of Pakistani heritage. She was unusual among participants in that she did not feel a sense of belonging to this city:

Extract 2: Priti, Pakistani woman, 20s

I barely go out so […] I haven't seen lots of places. It's more of home, shopping, takeaways, town maybe or just like park basically or uni that's it that's all I do. I mean people say that Manchester's really nice and it's beautiful […] but I haven't actually been so […] I can't exactly say how I like it if I don't know everything about it.

Her interview highlighted how she felt separate from the geographical Manchester community and this was not meaningful to her own identity. Her identity was as a daughter in a Muslim family, as opposed to a Mancunian and these two identities did not co‐exist for Priti, in the same way that they co‐existed for other participants of BAME origins. Notably, Priti did not identify with the positive aspects of Manchester that the other participants discussed and did not share the general sense that Manchester was a welcoming city. Instead she characterized the diversity of the city as segregated, rather than mixed. In reference to her own residential area, she reported: ‘*it's mostly Asians […] they're like, all Asians*’.

On the contrary, other participants, including those from BAME communities, felt that Manchester is a city historically known to welcome all races and cultures.

Extract 3: Mo, Pakistani man, 40s

I just think historically, we've always as Mancunians […] been here for the migrant communities. Whether it's war‐torn countries, we had Bosnians come here in big numbers you know […] we do a lot of memorials for Srebrenica in Manchester, huge Irish community, one of the oldest Irish dynasties are based half a mile from here […] We have Asian festivals, we have Jewish community here. It works very well.

Mo demonstrated his specific belonging (referring to himself as Mancunian) and knowledge of the diversity of the area, both historically and in the present day. Mo, as an ethnic minority community member, described how his own perception of Manchester is inclusive, and he later referred to his first‐generation immigrant mother as experiencing the same sense of positivity. The ethnic diversity of the city, while seen as a clear strength in relation to identity, was also something that made residents vulnerable if they were not connected to the wider community (as is the case for Priti) who viewed their lack of belonging as isolating and disenfranchising. This categorization of being a member or excluded from membership to this social category (i.e., being Mancunian) can have implications for how participants perceive the events and their response to them, which are discussed in the next two themes.

### Theme 2 ‘You've got a hug of people around you’: Coping in response to the attack

Theme 2 relates to how the community of Manchester took stock of what happened following the bomb through seeing it as an attack on the city as a whole, and how they coped collectively with the event. This process included a purposeful ‘coming together’ in some way following the bomb which strengthened feelings of belonging and identity. This theme consists of three sub‐themes: first *taking stock of the event*, which is the cognitive precursor to action; second, purposeful action in the form of *helping others* following the bomb; third, participation in *collective grieving and tributes*.

#### Taking stock of the event

Participants discussed how the existing identity of Manchester was so strong that it was inevitable that the attack would be experienced collectively by residents, in that ‘everyone’ was affected. Although they were not directly involved in the events, participants reported experiencing a shared sense of shock and disbelief as well as fear and uncertainty. The perception that the bomb affected the wider city of Manchester and Mancunians as a whole was evident through talk about how they collectively came together and made sense of what they were experiencing:

Extract 4: Ken, white British male, 20s

I think it has definitely added to this kind of togetherness that I experienced in Manchester when I came here this sort of tight‐knittedness […]. It definitely affected people the attack you know. I remember speaking to people in the days after and a lot of people were in shock, so I think when you're in that kinda state your response is just to do what you know, most people on your side are doing you know. We're all going to this, we'll all do that, so there's a kind of solidarity there.

Ken identified that community members were affected as a whole and experienced negative reactions together. Ken's use of the phrase ‘*most people on your side*’ makes it evident that he perceived the community as a collective source that can help reduce the threat of the event and provide strategies to respond to the bomb. He felt this was beneficial in uniting people from within the Manchester community (a sense of ‘*solidarity*’), when Manchester itself was the unifying context. The nature of the event in a specific geographical area that was a landmark in the city and familiar and proximal to many, enhanced this sense of threat.

In contrast, Priti (mentioned above in Theme 1) felt separate from the cohesiveness of Manchester, so viewed the event as something that increased her sense of victimization, vulnerability and fear within the wider Manchester community.

Extract 5: Priti, Pakistani woman, 20s

So, you have like a circle [gestures circle] and all the community basically inside and we were basically outside of the circle basically just like doing things but like no one actually knows to helping to just try and stay away from all the public basically.

Priti went on to illustrate who was inside the circle [‘*everyone else that weren't classed as terrorists*‘] and who was outside [‘*those that felt like they could be blamed, that they were being blamed and that they were at risk*‘], clarifying that she felt it was a dangerous time to be Muslim following the bomb. A sense of being ‘separated’ as a Muslim was relevant to her, and her reference to ‘we’ referred to her Muslim identity. Priti referred to her and her family feeling ‘scared’ in the city both prior to and after the Manchester Arena bomb on multiple occasions and her response was to withdraw further from the Mancunian community into the safe realm of family and home.

Thus, for Priti the threat does not derive only from the collective event itself (the bomb) but also the repercussions that the event would have on social cohesion and to her Muslim identity (her reason for staying away from the public). Furthermore, rather than turning to the community for help and support (to be discussed in theme 2.2) to reduce her appraisal of threat, she is instead left with no place for ‘help’ and ‘outside of the circle’.

#### Helping behaviour

For those who felt part of the community, community members reported purposeful, collective behaviour through *helping others* following the bomb. Most described myriad experiences of helping behaviour including practical, spiritual, emotional and psychological forms of assistance. This was felt as an expectation of City residents:

Extract 6: Bella, white, Eastern European woman, 20s

they would be actually there face‐to‐face, grieve together, cry together with the victims, put their flowers down, put their sadness there and they would all get along. It was going for weeks … every weekend … would be an hour that this particular, for the memory of the people, all the victims that passed away or have been injured in any way and … there were taxi drivers that were giving [lifts] there were people going like crazy to give food, to give blankets, to help … that showed the compassion of the people at the time and you know … it wasn't selfish.

Bella focussed on practical, care‐giving assistance to meet basic human needs emphasizing that it was other‐focussed rather than selfish, helping the direct victims but also other members of the community. The fact that the media emphasized the bomber as being from a Muslim background was relevant to some participants regarding their subsequent helping behaviour:

Extract 7: Nadia, Pakistani woman, 50s

They've [Muslims] been very visibly part of the community that's come together in the city centre and made efforts to go and say things there and be part of it, support it and some of our charities for so many days […] took water to the crowds and food to the crowds you know. That was very much coming from the Muslim community because they wanted to make a statement you know*…that* ‘*absolutely against all of this and we are not part of that, and our faith isn't part of that. We wanted people to hear*.’

Nadia spoke about the need to be seen and heard as positive, active members of the community, as a way of distancing themselves from the negative act of the bomber and managing their reputation.

#### Grieving and tributes

Participants reported collective forms of grieving such as leaving tributes and mass attendance at memorials, concerts and religious ceremonies. They reported a need for residents to physically come together to collectively grieve for those affected by the bomb, and the process of engaging collectively in some form of remembrance was generally reported by participants as cathartic. In trauma terms, these processes are a distinct form of support that help validate emotions and promote collective healing. Collective sharing of emotions in this way has been found to be a positive experience (Hopkins et al., 2016; Páez et al., 2007; Rimé, 2009).

One of the most significant tributes discussed by participants related to the symbol of the Manchester ‘worker bee’. Following the bomb, the symbol of the bee was used across social media and in various ways to denote solidarity with the city and pay tribute to the victims of the bomb. Participants viewed this as a fitting tribute to victims and discussed how the bee symbol was helpful to enhance a feeling of unity, as highlighted by Dave:

Extract 8: Dave, white British man, 50s

It does create a degree of unity so if you look like any tribe, if you have got a badge and you see lots of other people with the same badge there is a sense of unity there even with people you have never met. That does create some sense of community there. There did seem a sense of appropriateness that works as a symbol for Manchester […] yeah happy to be associated with that, happy to be associated with Manchester

Dave discussed how he felt that the purpose of the symbol was to be able create that instant connection with people of the same ‘tribe’ in the absence of actually knowing them personally. For Dave and other participants, a sense of cohesion and unity was brought about by using the symbol. It allowed people of different faiths and backgrounds to see they had a common group membership. More recently, the worker bee symbol has been combined with the Star of David in community and social media tributes as a show of solidarity following the Manchester synagogue attack on 2nd October 2025. This lends support to the symbol being viewed as an enduring sign of Mancunian identity and resilience.

While the interfaith links were reported by most participants as being strong in Manchester after the bomb, Priti did not share this positive sense of unity:

Extract 9: Priti, Pakistani woman, 20s

someone be racist only once since I've been here, so for the rest of time was totally fine, but since the bombing it's more … people give you weird looks or they'll look at you weird or they'll try and stay away from you, so it's [racism] basically increased since then.

Priti did not take part in any of the communal tributes as she felt the need to stay away for her own safety, maintaining the sense of perceived threat. In addition, unlike other participants, she felt that racism increased following the bomb. Her perception was supported by research that racist incidents often rise after Islamist terrorist attacks (NPCC, 10 Aug 2017; Hanes & Machin, 2014).

### Theme 3 ‘It takes the crap and makes it beautiful’ ‐ transforming for the future

Theme 3 follows from Mancunian identity as being meaningful (Theme 1) and coping collectively with the bomb (Theme 2) to the need to move beyond the event to look towards the future. This theme consists of three sub‐themes: *Restoration and Hopefulness*, *Active Belonging* and *Managing Divisions and Potential Conflict*.

### Restoration and hopefulness

Participants reported that over the course of the collective grieving process, a shared sense of recovery emerged. Participants often referred to visual symbols of hope and regeneration such as the worker bee. The 1996 IRA bombing of Manchester was also referenced, and in this context, a post‐box had the added importance of signifying the subsequent rebuilding of the city centre.

Extract 10: Julie, white British woman, 30s

I think that post‐box just was like yeah, get lost you know. We're still gonna be here yeah. I just think it was ace and it was so […] there was no verifiable reason for it to be wholly intact. So that was just like a really happy, hopeful thing … we rebuilt then and we rebuilt again. I mean in a lot of ways, they're different in that the IRA bomb, the physical destruction to the city centre was just terrible, but the Arena was easily rebuilt.

Julie made a point of relating the post‐box to defiance against the terrorist act of destruction and any aims it had to destroy the city. She indicated that she had thought rationally about how the post‐box had survived the destruction, yet could not find a reason, which helped her to elicit positive feelings of hopefulness.

The symbol of the worker bee was also used to link together aspects of hopefulness, transformation and creativity:

Extract 11: Fliss, white British woman, 40s

it's an identity and not with the negative. it's not an identity with the bomb, it's an identity with being a part of this community and being proud and it's something that people wear with pride… I actually found it interesting that you know, a worker bee is actually in reality, it's quite a dull, unattractive thing. But Manchester has reinvented it and transformed it into something glorious and fat and yellow and brightly coloured and beautiful and yeah, that's what Manchester does. It takes the crap and makes it beautiful.

Fliss' use of visual description regarding the symbol reflects how she (and Manchester) conceptualized being able to transform something negative into something positive. For Fliss, the post‐box and the bee symbol were linked with a sense of hopefulness about the future and a need to look towards the future instead of feeling sad about the past. Fliss was able to identify that regeneration in Manchester was possible and provided hope to the community.

#### Active belonging

The importance of attending community‐focussed meetings and rallies to promote long‐term community change was identified as something that assisted the community to move on from the bomb, with a positive sense of collective purpose. This was linked to the sense of belonging that participants felt with each other and with Manchester. It was felt that this related to a need to do something that went beyond practical helping behaviour at the time of the bomb to promote peace and inclusion in the city for the future.

Amira, a woman of Libyan ethnicity in her 30s, expanded upon the context of this for her Libyan community in the following extract:

Extract 12: Amira, Libyan woman, 30s

I had loads of people contact me who never contacted me before, never been engaged in community activities from the Libyan communities, specifically when they found out that it was a Libyan who did this … that became a regular thing to meet up and think about what the response from the Libyan community was going to be and whether it was more respectful to just join other things that were happening [in Manchester] rather than stand as a single community as if we were speaking for everyone, because obviously it wasn't representative of everyone … and that was the only surprise for me, I mean I knew there would obviously be responses from the council and various community groups but I wasn't expecting that the Libyan community was gonna actually kind of respond in that way so that was interesting.

Amira discussed how the Libyan community were conscious of the bomber being from their community, so they wanted to respond which surprised her, as they were not previously engaged in community meetings led by local officials. Members of the Libyan community chose to take part in fund‐raising activities as part of the community (BBC News, 2017). When asked to consider why she thought they wanted to get involved, she added.

Extract 13: Amira, Libyan woman, 30s

it feels like maybe you do have to prove something and I think that's how a lot of them [other members of the Libyan community] felt. For me it felt OK because I'd always been engaged in that kind of stuff, whereas for them I think they felt they had to do stuff that they wouldn't necessarily normally do, as if they needed to prove that they were good people, that they were part of the community that was going to be responsive and positive.

Amira spoke about the Libyan community challenge being the feeling that they had to ‘prove’ they shared the characteristics of the ‘good people’ who were part of wider community taking positive action. Engagement appeared to be related to the need to identify with the wider community, rather than just their ethnic identity at that time.

#### Managing divisions and potential conflict

Unity across groups was perceived as important by several participants. For example, extract 7 speaks to the efforts made by the Muslim community to distance itself from the bomber and unite with other communities. Similarly, Christian, other Muslim and Jewish participants spoke about the impact of religious faiths being seen to come together in response to the bomb. It was perceived as a necessary response, given that the bomber was from a Muslim background, and this was something that was being presented as a significant feature through the media, so it could not be ignored. The extract below indicates how this ‘coming together’ of religions could manifest.

Extract 14: Dave, white British Christian male, 50s

the vicar at (name) Church in (location) went down to his local Mosque and just stood there and chatted to people to show that there was a solidarity […] He wanted to make a stand that that didn't mean that the Christian community blamed the Muslim community […] Clearly, he was responding to a fear within the Muslim community, and he thought that was an important stand to make, to show love, concern, neighbourliness and did so quite publicly.

Dave highlighted how the Christian vicar wanted to be seen to support the Muslim community from a collective Christian perspective, not only as something he did individually. It is clear that Dave perceived it to be important that a Christian leader made this public demonstration of solidarity at the time.

When asked how non‐religious community members were included as part of this spirit of ‘coming together’, other participants identified very clear collective examples such as a poem about Manchester being read publicly by the poet, Tony Walsh as well as the spontaneous singing of ‘Don't look back in anger’, a song by Mancunian band Oasis. While participants perceived many positive examples of community engagement following the bomb, there was a view that those with less power in the community felt that there was no point in engaging, as it was not something that could elicit change. Mary, discussed this below:

Extract 15: Mary, white British woman, 30s

You do need to have somebody with some power deeply engage with those communities get to know them and the reason I say with some power is they also have money which enables opportunities […] The problem isn't with the community, the problem is [long pause] Manchester's structure isn't connecting and empowering some of the most vulnerable communities […] the values of this city doesn' stand for that, that's not what Manchester's about so it jars with what we say we do […] I believe Manchester's values are… is that it, it doesn't want any community to feel disempowered it says it doesn't so it needs to do more.

Mary felt that low levels of engagement between socially disadvantaged communities and those in power was an intrinsic problem at the heart of Manchester. Mary had previously discussed that the city's identity was of connection and belonging, so for her, the local government needed to strive to ensure this was achievable for all parts of the community. Otherwise, power imbalances in communities were perceived as directly tied to notions of safety, fear and danger. For Mary, the sense of ‘otherness’ that exists between sub‐communities is not conducive to a healthy society and could lead to powerlessness and fear.

Theme 3 identified how such hopefulness and acceptance drove collective action and highlighted how such collective action further strengthened community members' sense of place identity. The recovery of the city was seen as something inherent in the existing identity of the community. However, participants also discussed how important it was to maintain community cohesion and how part of this responsibility is addressing marginalization that could lead some communities to be excluded from the process of recovery.

## DISCUSSION

The analysis of these interviews with 18 Manchester residents reveals how the identity of being Mancunian shaped their individual and collective responses to the Arena bomb. More specifically, it shaped how they appraised the threat of the events, coping responses and how they built resilience. In line with previous research on collective trauma (Muldoon et al., 2019), their shared identity as Mancunian meant that they collectively experienced negative effects from the bomb, even though they were not directly involved. Their accounts of their collective coping with the aftermath of the bomb drew heavily on the city's history and character. The solidarity between faith groups was taken to reflect a strong norm of diversity, while the expectation of recovery from the events was reported to stem from past experience of unity and overcoming adversity.

Moreover, these perceptions were reported to feed forward into collective behaviour, such that the shared sense of identity led to a coordinated collective attempt to help others, even across different subpopulations, and to symbolically and practically overcome the challenge. Conversely, where these understandings of Manchester were absent among marginalized populations, participants reported or noted a sense of increased exclusion, isolation and disempowerment.

These findings speak directly to the SIMCR model of community resilience (Drury et al., 2019). As noted above, while SIMCR and other approaches to understanding community resilience to disasters acknowledge the likely effects of pre‐existing social relationships upon community responses to disasters (Carter et al., 2020; Drury et al., 2019), SIMCR primarily focuses on those directly affected by the events and upon the social identity emergent from the disaster itself. Our study indicates that the meanings associated with long‐standing place‐based identities can have great importance for how residents cope with disasters. At a most basic level, sharing such a place‐based identity means that an aversive event affecting some of the population can be experienced indirectly by all members. In other words, the psychological impact of disasters extends beyond those immediately affected and reaches all of those sharing the relevant social identity.

Second, the content of identity matters. For most of our participants, the understanding of Mancunian identity as diverse and resilient provided psychological resources with which to construe the events (primary appraisal), ascertain the level of collective coping resource (secondary appraisal) and coordinate their joint response. While the relationship between identity content and coping response has previously been noted in passing in relation to the ‘blitz spirit’ response among survivors of the London Bombs of 2007 (Drury et al., 2009) in which the historic experience of Londoners was used to interpret and respond to the attack, it has not been considered as an element of identity‐based disaster responding in its own right.

The converse of this finding is equally important. For the single participant (member of a marginalized group) who lacked any sense of shared Mancunian identity, the events were reported to result in further exclusion and discrimination. This in turn was recognized by other participants who acknowledged that the inclusive nature of the identity‐based collective coping response extended to some communities, but not others and emphasized how important it was to overcome these divisions. This suggests that understanding the different ways in which individuals relate to their local identities, and their group memberships, is key to appreciating the boundaries of community responses to disasters. Those who feel excluded from the shared identity of the locale may find themselves subject to further ‘social curse’ effects (Këllezi et al., 2009; Këllezi & Reicher, 2012) during a disaster which may deprive them of the collective coping resources afforded to other group members.

The practical implication of this work for the SIMCR approach to disaster resilience is that to predict how a local community (of whatever scale) will respond to a disaster, it is necessary to capture beforehand how residents themselves understand their identities (Carter et al., 2020). From the current case study, the most important aspects would appear to be the sense of unity or division in the area, the feeling of collective efficacy in dealing with unforeseen challenges and the relationships between people and place. This also suggests that this dynamic can and should be harnessed to overcome adversity. If identities of residential areas can be effectively represented and enacted as united, cohesive and inclusive, local communities should fare better than those with identities associated with division and conflict.

More generally, the contribution of these findings to the Social Identity Approach to Health is twofold. First, this work contributes to a growing body of evidence attesting to the importance of residential community in determining the health and well‐being of residents (McNamara et al., 2013, 2021). Second, the novelty of the present study lies partly in the greater scale of the residential identity (to date, this is the first evidence of the ‘social cure’ at city level) and also in the importance of the geographical and historical dimensions to its residents. This suggests that it may be fruitful in future to explore the relationships between people and place identities when investigating the protective characteristics of locality‐based identities.

There are of course a number of limitations to the study which need to be acknowledged. These interviews were retrospective accounts of the events and responses to the Manchester bomb rather than contemporaneous evidence. No doubt participants will subsequently have been exposed to a multitude of different representations of the events from many different quarters which may differ from their original experiences and perceptions and could have influenced their recollection and recounting. However, we would argue that this collective sharing of experiences and narratives of the event over time is an essential part of collective coping and that triangulation of retrospective accounts can help evidence these group dynamics (Binder et al., 2015; Chamlee‐Wright & Storr, 2011). Nonetheless we would recommend that future research adopting a prospective approach to capturing community identity dynamics ahead of disasters and emergencies would be better placed to capture these longer term identity effects on community resilience.

A more challenging limitation is the sample size and diversity which cannot be argued to span the full diversity of Manchester's population. Socially isolated and marginalized communities are typically under‐represented in this type of research and our participant, Priti is the sole representative of those who feel excluded from mainstream society on the basis of race or class. At best, this analysis offers a glimpse into the ‘social curse’ dynamics of alienation and marginalization among those who are most socially vulnerable to the adverse effects of natural and man‐made disasters. Again, future research into disaster preparedness would benefit from outreach work to identify those communities and individuals who feel excluded from their local communities as, in the event of a disaster, these are more likely to miss out on collective coping responses. Our work suggests that a fuller understanding of how to operationalize social identity to include marginalized group members could provide resilience to the socially divisive effects of terrorist attacks and other disasters.

## AUTHOR CONTRIBUTIONS

**Helen Hart:** Conceptualization; investigation; writing – original draft; writing – review and editing; visualization. **Clifford Stevenson:** Supervision; writing – original draft; visualization; writing – review and editing. **Blerina Kellezi:** Writing – review and editing; supervision; visualization; writing – original draft.

## CONFLICT OF INTEREST STATEMENT

The authors have no conflicts of interest to declare.

## Supporting information


Data S1:

## Data Availability

Research data are not shared as it relates to locally identifiable human research participants.
