# Supplementary material for: Manchester stands united: Place‐based identity facilitates resilience in the aftermath of a mass emergency
Source: Br J Soc Psychol. 2026 Feb 17;65(2):e70056. doi: 10.1111/bjso.70056 (PMC12914091; doi:10.1111/bjso.70056)
Supplement: Supplementary file 1 — Data S1: [file BJSO-65-0-s001.docx]

**Supplementary Material**

**Interview Schedule**

1. Intro. Describe how you fit in your local community – your connections, feeling settled, feeling part of Manchester?
2. Identity *- Explain social identity – ways of seeing ourselves as belonging to certain groups, as being like them so feeling part of them.* What are your own groups and where do you fit in. Why do you feel like that?
3. Bomb – *Introduce the research context.* Thinking of yourself at the time, what was the impact personally, what did you see in how family were effected, same question for friends, local community in general, Manchester, Identity groups?
4. Passage of time – as time went on over days, weeks, months, were there any changes in how you processed the above over time? Same question for Manchester over time? What helped and hindered with this?
5. Now – How do you perceive your personal and community strengths and vulnerabilities since the bomb? What local changes have you seen?
6. Future – what can help Manchester now, regarding what happened? What can help into the future? What could other communities learn from how Manchester responded to the bomb then and following the bomb? What could other communities do better?
7. Summary wrap-up – revisit points. Summarise and ask for views about other relevant interviewees perspectives – *some participants thought that xxxx what do you think?*
